# Molecular age estimation based on posttranslational protein modifications in bone: why the type of bone matters

**DOI:** 10.1007/s00414-023-02948-9

**Published:** 2023-01-17

**Authors:** Lisa König, Julia Becker, Alexandra Reckert, Stefanie Ritz-Timme

**Affiliations:** Institute of Legal Medicine, University Hospital Düsseldorf, 40225 Düsseldorf, Germany

**Keywords:** Age estimation, Pentosidine, Aspartic acid, Bone

## Abstract

Age-at-death estimation is of great relevance for the identification of unknown deceased individuals. In skeletonised corpses, teeth and bones are theoretically available for age estimation, but in many cases, only single bones or even only bone fragments are available for examination. In these cases, conventional morphological methods may not be applicable, and the application of molecular methods may be considered. Protein-based molecular methods based on the D-aspartic acid (D-Asp) or pentosidine (Pen) content have already been successfully applied to bone samples. However, the impact of the analysed type of bone has not yet been systematically investigated, and it is still unclear whether data from samples of one skeletal region (e.g. skull) can also be used for age estimation for samples of other regions (e.g. femur). To address this question, D-Asp and Pen were analysed in bone samples from three skeletal regions (skull, clavicle, and rib), each from the same individual. Differences between the bone types were tested by t-test, and correlation coefficients (*ρ*) were calculated according to Spearman. In all types of bone, an age-dependent accumulation of D-Asp and Pen was observed. However, both parameters (D-Asp and Pen) exhibited significant differences between bone samples from different anatomical regions. These differences can be explained by differences in structure and metabolism in the examined bone types and have to be addressed in age estimation based on D-Asp and Pen. In future studies, bone type-specific training and test data have to be collected, and bone type-specific models have to be established.

**Supplementary Information:**

The online version contains supplementary material available at 10.1007/s00414-023-02948-9.

## Introduction

In the identification of human remains, age estimation is of key importance. To date, a great repertoire of morphological, molecular, and physicochemical methods is available for age estimation [1–4]. The selection of the most suitable method in a specific case depends on the type and condition of the available samples. The most relevant entity of sample type is bone. The available material for analysis can range from an entire skeleton to a sample of only one isolated bone or bone fragment.

In adult age, a sufficiently accurate age estimation based on morphological findings may be difficult or even impossible, e.g. in cases of isolated bones or bone fragments. The analysis of bone samples via molecular methods based on posttranslational protein modifications and DNA methylation opens up new possibilities for age estimation.

Protein-based approaches for molecular age estimation can be based on the age-dependent accumulation of D-aspartic acid (D-Asp) and pentosidine (Pen) in long-living proteins of various tissues [5–14].

The accumulation of D-Asp is the result of spontaneous conversion of L-aspartic acid into its D-form at c. 37 °C body temperature (for details, see [15, 16]). The kinetics of this process depends on the structure of the affected proteins; the fewer steric hindrances hinder the process, the faster the conversion of L-residues in their D-form may occur. That means that every protein has its own kinetics of D-Asp accumulation. The process is temperature dependent. After death, it ceases in lower ambient temperatures (as compared to an in vivo body temperature of 37 °C). Given forensically relevant post-mortem intervals of up to several decades and no extreme surrounding conditions (as in the case of burnt bodies), no relevant impact of a post-mortem conversion of L-Asp into D-Asp on age estimation is to be expected [17].

The Pen is an advanced glycation product that accumulates in an age-dependent manner in long-living proteins under healthy conditions (for details, see [18]) and seems to be extremely stable after death; Mahlke et al. (2021) report plausible age estimates based on Pen in dentine even after millennia [19]. Since the formation of a Pen depends on—among other factors—protein structure, every affected protein has its own kinetics of Pen accumulation. Pathological metabolic conditions such as long-lasting hyperglycaemic states or renal failure may result in elevated Pen levels and thus in false high age estimates [20–24]. A combined analysis of D-Asp and Pen (and DNA methylation) is recommended to detect such errors caused by metabolic diseases [14].

D-Asp and Pen accumulate in an age-dependent manner only in long-living proteins. Highly bradytrophic and homogenous tissues are optimal for the application of these methods. Especially dentine is an ideal tissue for age estimation based on D-Asp and Pen [25, 26]. Bone tissue, however, is neither bradytrophic nor homogenous, but it does contain long-living proteins like osteocalcin [1, 27]. If such long-living bone proteins are purified, age estimation based on posttranslational protein modifications may be very accurate [1, 27]. The analysis of such purified protein samples is highly sophisticated, and the applicability of such methods in forensic practise is therefore limited. However, bone tissue contains long-living proteins in such high concentrations that the detection of an age-dependent accumulation of D-Asp and Pen is possible even if the long-living proteins are not purified [2, 28, 29]. Admittedly, the accuracy of age estimation based on D-Asp and Pen in non-purified bone samples (total protein or non-collagenous samples) is much lower compared to purified bone protein or dentine samples. Nevertheless, accuracy appears to be high enough for an application of these molecular methods to non-purified bone and seems to be superior to morphological methods (at least in adult age), especially if applied in a combined model [3, 14].

The potential of these protein-based approaches for age estimation by analysis of bone samples has to be further explored. One important question yet to be answered is the impact of the anatomical origin of the analysed bone sample; this aspect has not yet been systematically investigated. So far, most studies on bone have focused on samples of skull or femur [1, 5, 29, 30], and it is unclear if data from one of these skeletal regions can be used for age estimation by analysis of another, e.g. a rib sample.

An impact of the type of bone sample on age estimation based on D-Asp and Pen can be expected since different types of bone vary in tissue structure and kinetics of turnover [31–33]. If not purified bone proteins, but total tissue samples or extracted protein fractions (e.g. the non-collagenous protein fraction after acid extraction) are analysed, the composition of these mixed protein samples is of relevance for the results of D-Asp and Pen analysis. Somewhat simplified, one could assume the following: the more long-living proteins in a sample, the higher the D-Asp and Pen concentrations. Accordingly, D-Asp and Pen concentrations can be expected to vary in samples of bones from different anatomical origins.

A better understanding of the impact of the type of bone on age estimation based on D-Asp and Pen is of utmost importance for forensic casework as well as for the planning of further research.

Therefore, we tested the hypothesis that the anatomical origin of bone samples has an impact on age estimation based on D-Asp and Pen by analysing and comparing bone samples from skull, rib, and clavicle of 58 individuals.

## Material and methods

### Bone samples

Samples from the skull, one clavicle, and one rib of 58 individuals with known ages between 0.21 and 94 years were collected during routine forensic autopsies. Individuals with known diabetes mellitus or advanced kidney disease were excluded from Pen analyses. The post-mortem intervals were between approx. 5 h and 8 days. Samples for all three bone types were available for 51 individuals for at least one parameter; in 6 cases, only skull samples and in one case, only clavicle samples could be analysed. In some cases, the Pen concentrations were under the detection limit. Table 1 in the supplementary material gives an overview of the available material and data (Table 1).

### Preparation of bone samples

From each individual, samples from the skull, one clavicle, and one rib were analysed. Skull samples were taken from the left parietal bone, close to the usual saw cut for opening the skull during autopsies, clavicle samples were dissected from the medial half of the left clavicle, and rib samples were collected from the middle third of the fourth rib on the left-hand side.

Soft tissue and the cancellous parts of the bone samples were removed mechanically. The bone samples were sawn into approx. 1 × 1 × 0.5 cm large fragments and pulverised by Ika Tube Mill Control (17,000 rpm). The resulting powder was washed in distilled water, 15% sodium chloride, 2% sodium dodecyl sulphate, and ethanol/ether (vol. 3:1), respectively, lyophilised and stored at − 20 °C until further analysis.

### Determination of the D-Asp content by analysis of D- and L-aspartic acid

The D-Asp content of bone was determined in total protein (TP) samples as well as in the non-collagenous bone fraction (NCP). The NCP was prepared by acid extraction: 7 ml of 0.6 N HCl was added to 200 mg of bone powder, the sample was shaken for 15 min, centrifuged, and an aliquot of 1.4 ml of the supernatant was dried.

Bone powder samples (TP and NCP) were hydrolysed for 6 h with 1 ml of 6 N HCl at 100 °C.

D-aspartic acid and L-aspartic acid were analysed by high-performance liquid chromatography (HPLC) as described by Becker et al. 2020 [14] with minor modifications (shortened gradient). Samples were dissolved in 1 ml sample buffer (0.01 M HCL with 1.5 mM sodium azide and 0.03 mM L-homo-arginine). For HPLC analysis, a C18 column from Thermo Scientific (Hypersil BDS C18, 250 × 3 mm, particle size 5 μm) was used as the stationary phase. The mobile phase included eluents A (23 mM sodium acetate, 1.5 mM sodium azide, and 1 mM EDTA) and B (92.3% methanol, 7.7% acetonitrile). The amino acid enantiomers were detected by a gradient over a period of 49 min at a constant flow rate of 0.56 ml/min. Amino acids were detected at an excitation wavelength of *λ* = 230 nm and a detection wavelength of *λ* = 445 nm. D- aspartic acid and L-aspartic acid) were identified by their retention times.

The D-Asp content was expressed as ln ((1 + D/L)/(1 – D/L)), with D = D-aspartic acid and L = L-aspartic acid).

### Determination of the Pen content

The Pen content of the bone samples (TP) was analysed by HPLC as described by Greis et al. [34], with several modifications (introduction of a solid phase extraction, shortened gradient in HPLC). A total of 100 mg of bone powder were hydrolysed with 1 ml of 6 N HCl at 110 °C for 18 h. After drying, 1 ml of 0.01 M heptafluorobutyric acid (HFBA) was added. The solution was filtered through syringe filters (Ø 25 mm and 0.45 µm pore diameter), and solid phase extraction was performed (Phenomenex, Strata-X 33 µm Polymeric Reversed Phase). The dried samples were dissolved in 200 µl of pyridoxine-HFBA buffer. A total of 50 µl of each sample were injected into the HPLC system. The stationary phase was a semi-preparative column (Onyx™ Monolithic Semi-PREP C18, 100 × 4.6 mm) by Phenomenex. A linear gradient of acetonitrile (eluent B) and 0.1% HFBA in HPLC water (eluent A) was used as mobile phase with a flow rate of 1 ml/min over a period of 37 min, an extinction/emission wavelength of 335/385 nm for detection of pentosidine. Pen was identified by its retention time. A pentosidine standard (pentosidine 0.03303 nmol/ml in 0.01 M HFBA, Cayman Chemical) was used to establish a calibration curve.

### Statistics

Spearman correlation coefficients (*ρ*) were calculated for both parameters (D-Asp and Pen) and all three types of bone. To test whether the D-Asp and Pen contents differ between the types of bone, *t*-test was performed for dependent samples. A *p*-value < 0.05 was considered significant.

## Results

### D-Asp in samples of different types of bone

**Fig. 1:**
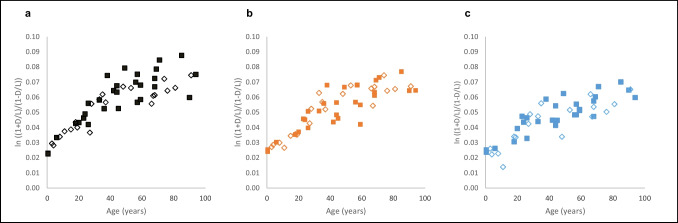
D-aspartic acid (D-Asp) content (as ln [(1 + D/L)/(1 – D/L)]; D= D-aspartic acid; L= L-aspartic acid) in total protein (TP) of female (◇) and male (■) bone samples, related to the age at death: **a** skull; *n* = 50 (21 females, 29 males), **b** clavicle; *n* = 50 (21 females, 29 males), and **c** rib; *n* = 46 (18 females, 28 males)

**Fig. 2:**
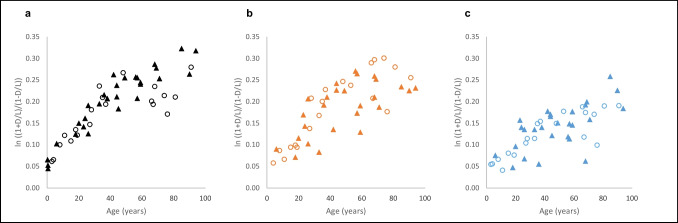
D-aspartic acid (D-Asp) content (as ln [(1 + D/L)/(1 – D/L)]; D= D-aspartic acid; L= L-aspartic acid) in ln [(1 + D/L)/(1 – D/L)] in non-collagenous total protein (NCP) of female (○) and male (▲) bone samples, related to the age at death: **a** skull; *n* = 49 (20 females, 29 males), **b** clavicle; *n* = 46 (20 females, 26 males), and **c** rib; *n* = 47 (20 females, 27 males)

All types of bone exhibited an age-dependent accumulation of D-Asp in TP samples as well as in the non-collagenous NCP fraction (Figs. 1a–c and 2a–c). The scattering of data increased significantly with increasing age; this is most obvious in the skull and clavicle samples; by contrast, they exhibit a very strong relationship between D-Asp and age in younger ages.

In the TP samples, the correlation of D-Asp concentration and age was very similar in all bone types analysed (clavicle: *ρ* = 0.85, skull: *ρ* = 0.84, and rib: *ρ* = 0.84). In the NCP samples, the correlation was strongest in skull samples (*ρ* = 0.85), followed by the clavicle (*ρ* = 0.76) and rib samples (*ρ* = 0.72).

*T*-test of dependent samples revealed statistically significant differences of D-Asp concentrations in skull v. in rib (*p* = 0.000000000014), in clavicle v. in rib (*p* = 0.00000017), and in skull v. in clavicle samples (*p* = 0.0000399).

Due to the low number of individuals, differences between female and male individuals could not be statistically tested; as shown by the highlighted female and male samples in Figs. 1a–c and 2a–c, there was no clear indication of relevant differences between the sexes.

### Pen in samples of different types of bone

**Fig. 3:**
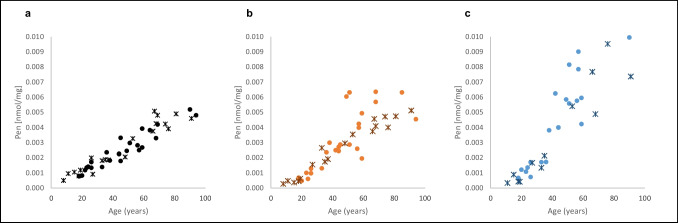
Pentosidine (Pen) concentration [nmol/mg] of female (**ж**) and male (●) bone samples, related to the age at death: **a** skull; *n* = 45 (20 females, 25 males), **b** clavicle; *n* = 43 (18 females, 25 males), and **c** rib; *n* = 37 (13 females, 24 males)

Pen concentrations accumulated with increasing ages in all examined types of bone. In the rib samples, Pen concentrations exhibit very high values in older ages and a much higher scattering, as compared to the other bone types (Fig. 3a–c). The clavicle and rib samples showed a markedly increasing scattering of data with increasing ages.

The relationship between Pen concentration and age was closest in skull samples (*ρ* = 0.95), followed by rib (*ρ* = 0.93) and clavicle samples (*ρ* = 0.90).

T-test of dependent samples revealed significant differences in Pen concentrations in skull v. in rib (*p* = 0.00013) and in clavicle v. in rib samples (*p* = 0.00018). There was no statistically significant difference in Pen concentrations in skull v. in clavicle samples (*p* = 0.07).

Again, differences between female and male individuals could not be statistically tested (due to the low number of individuals); the highlighted female and male samples in Fig. 3a–c also do not indicate clear differences between the sexes.

## Discussion

For the first time, D-Asp and Pen were analysed in different types of bone in direct comparison (different types of bones from each individual included in the study, all analyses in one lab). The results of these analyses confirm the hypothesis that the type of bone used has an impact on age estimation based on D-Asp and Pen.

For the interpretation of the presented data, it is of particular importance to understand the influence of the protein composition on the D-Asp and Pen concentrations in a mixed protein sample (TP and NCP samples); in such mixed samples, D-Asp and Pen levels are summary values consisting of D-Asp and Pen concentrations of every single protein in the sample. Each of these proteins has its own kinetics of D-Asp and Pen accumulation, especially depending on protein structure and metabolism (usually: no accumulation in proteins with very high turnover, low accumulation in proteins with slow turnover as well as in proteins with a complex structure and steric hindrances, and fast accumulation in long-living and small proteins [1]). Therefore, changes in the protein composition of a sample will have strong effects on the D-Asp and Pen concentrations in mixed protein samples.

*T*-test for dependent samples revealed significant differences between all types of bone analysed and all parameters examined, except for Pen in skull and clavicle samples. Differences concern both the kinetics of accumulation of D-Asp and Pen and the scattering of data.

These findings can be explained by differences in structure and metabolism in bones from other anatomical sites, resulting in varying protein compositions of the samples. The rib samples exhibited the most striking differences (especially for Pen) as compared to samples of skull and clavicle. This may be explained by a possibly higher rate of remodelling due to individual load and higher stress in ribs [35–37] as well as by the bone structure of the ribs with a high proportion of cancellous bone that possibly could not be removed in total.

A further explanation for the observed differences in the accumulation of D-Asp and Pen in the bone types examined may be variations in tissue ageing at a molecular level. The significantly wider scattering of data with increasing age in all bone types is typical for many biomarkers of ageing and due to an increasing destabilisation of the tissues and their functionality during ageing. Bone structure and metabolism are changing with increasing age; this process may result in loss of bone mass, decreasing thickness, and osteoporosis [31–33, 38, 39]. These structural and metabolic changes of bone with age may cause significant changes in the protein composition of bone samples, resulting in a significantly wider scattering of D-Asp and Pen concentrations with increasing age in mixed protein samples. It can be assumed that the effect of ageing processes on bone proteins varies in bones from different skeletal sites, and this might be another cause for the varying scattering patterns of data for TP and NCP samples of different bone types.

A stronger or weaker correlation of D-Asp and Pen with age has a direct impact on the accuracy of age estimation by these approaches. It was not the aim of this study to establish definitive models for age estimation, and we did not investigate an independent test sample to determine the errors of age estimation for the different bone types. Nevertheless, our data allow the conclusion that different errors in age estimation are to be assumed for different bone types.

## Conclusions: what do the presented data mean for age estimation based on D-Asp and Pen in bone?

D-Asp and Pen levels in skull, clavicle, and rib samples differed significantly from each other. Therefore, the results of age estimation based on D-Asp or Pen in samples from one specific bone type may become significantly worse if training data from other bone types are used (e.g. rib sample—skull training data). Optimal and valid results can only be expected if age estimation can be based on a bone type-specific model. Efforts to establish such bone type-specific models should be made to address different kinetics in age-dependent accumulation of D-Asp and Pen as well as different errors in age estimation.

The presented data confirm an age-dependent accumulation of D-Asp and Pen in bone samples that can be used for age estimation if sufficient training data are available for the type of bone to be analysed. Skull and clavicle samples appear to reveal more precise results than rib samples. The analysis of more than one bone type and more than one parameter may be useful if multivariate models based on training data for all bone types are available.

In principle, the use of multivariate approaches that use information from different biological contexts by including diverse parameters is recommended to address the significantly lower accuracy of age estimation in older ages as well as problems with confounding factors (e.g. long-lasting hyperglycaemic states or renal failure for Pen) [14].

## Supplementary Information

Below is the link to the electronic supplementary material.

Supplementary file1 (XLSX 20 KB)

## Data Availability

Not applicable.
